# X-ray structures of two proteins belonging to Pfam DUF178 revealed unexpected structural similarity to the DUF191 Pfam family

**DOI:** 10.1186/1472-6807-7-62

**Published:** 2007-10-01

**Authors:** Rajiv Tyagi, Stephen K Burley, Subramanyam Swaminathan

**Affiliations:** 1 Biology Department, Brookhaven National Laboratory, Upton, New York 11973, USA; 2 SGX Pharmaceuticals, Inc., San Diego, California 92121, USA

## Abstract

**Background:**

Pfam is a comprehensive collection of protein domains and families, with a range of well-established information including genome annotation. Pfam has two large series of functionally uncharacterized families, known as Domains of Unknown Function (DUFs) and Uncharacterized Protein Families (UPFs).

**Results:**

Crystal structures of two proteins from *Deinococcus radiodurans *and *Streptomyces coelicolor *belonging to Pfam protein family DUF178 (ID: PF02621) have been determined using Selenium-Single-wavelength Anomalous Dispersion (Se-SAD). Based on the structure, we have identified the putative function for this family of protein.

**Conclusion:**

Unexpectedly, we found that DUF178 Pfam is remarkably similar to Pfam family DUF191 suggesting that the sequence-based classification alone may not be sufficient to classify proteins into Pfam families.

## Background

Pfam is a comprehensive collection of protein domains and families, with a range of well-established information including genome annotation. Each family in Pfam is represented by two multiple sequence alignments and two profile-Hidden Markov Models (profile-HMMs) [1]. Pfam has two large series of functionally uncharacterized families, known as Domains of Unknown Function (DUFs) and Uncharacterized Protein Families (UPFs). DUFs are families that have been created by Pfam whereas UPFs are those created by Swiss-Prot and added to Pfam [1]. The Protein Structure Initiative-2 has undertaken the task of structurally characterizing all Pfam families that have no structural representation. The Pfam protein family DUF178 (ID: PF02621) consists of 61 proteins of unknown function, 59 from bacteria and 2 from archaebacteria [2]. Herein, we report the first crystal structures of DUF178 family members, including Q9RXE3 from *Deinococcus radiodurans *and Q9L0T8 *Streptomyces coelicolor *and show that they are remarkably similar to Pfam family DUF191.

## Results and discussion

**Figure 1:**
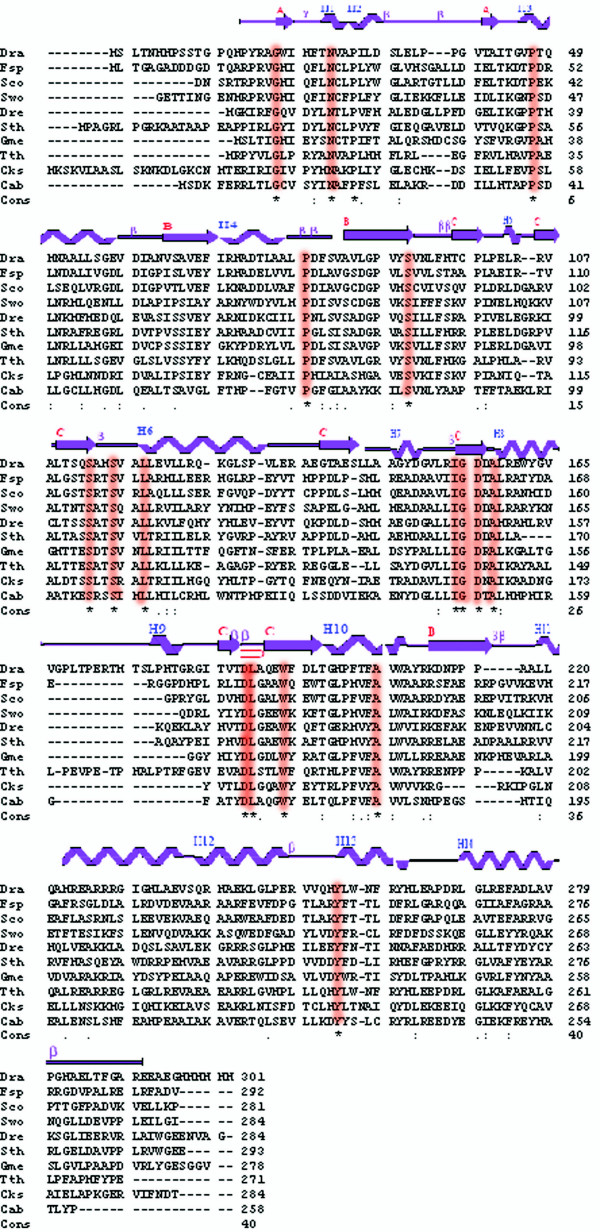
Multiple sequence alignment of DUF178 from various organisms. The residues highlighted in orange are the conserved residues (*). The abbreviations for organism names are as follows: Dra; *Deinococcus radiodurans (10093b)*, Fsp; *Frankia sp*. CcI3, Sco; *Streptomyces coelicolor (10093f)*, Dre; *Desulfotomaculum reducens*, Sth; *Symbiobacterium thermophilum*, Gme; *Geobacter metallireducens*, Tth; *Thermus thermophilus*, Cks; *Candidatus Kuenenia stuttgarti*, Cab; *Chlamydophila abortus*. The secondary structural elements for 10093b (residues 16 to 285) are given at the top of alignment.

**Figure 2:**
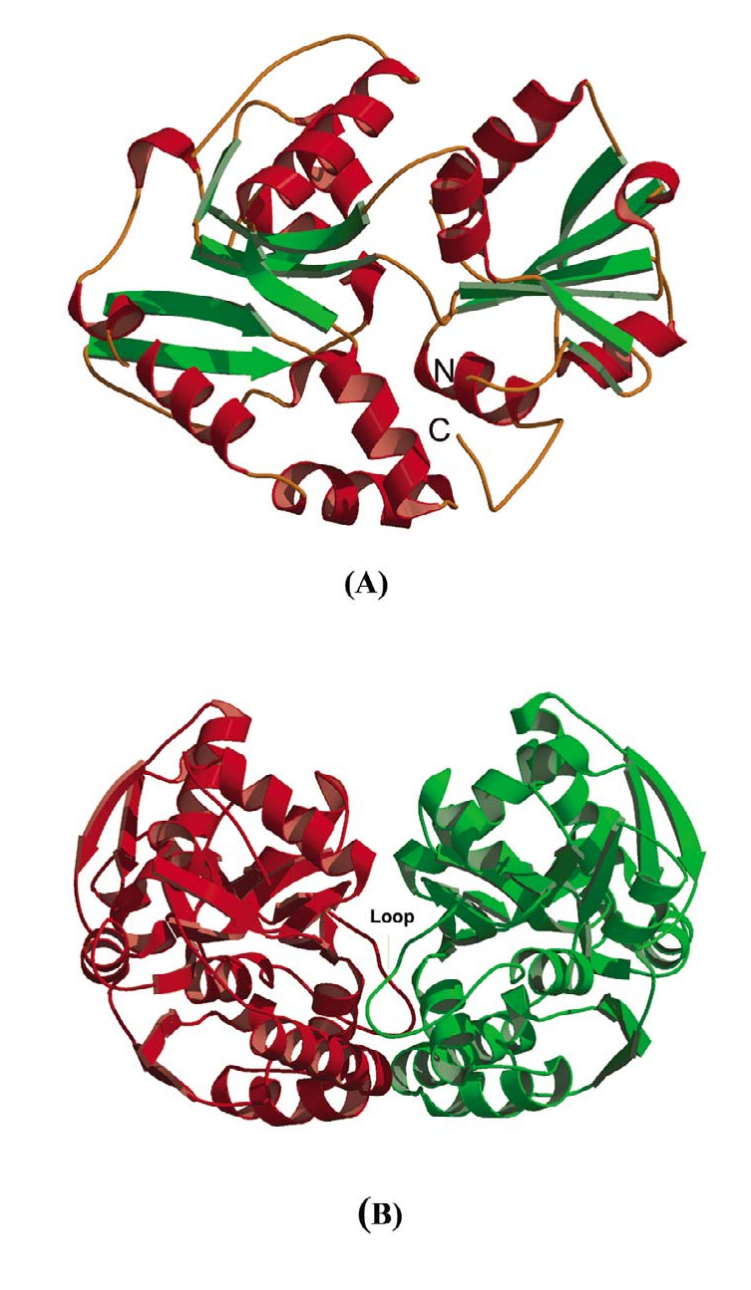
**(A) **Monomer of 10093b showing both N- & C-terminal domains. The 10093f monomer has the same fold. (B) Asymmetric unit of 10093b showing tightly packed dimer. Loop responsible for dimerization is labeled.

**Figure 3:**
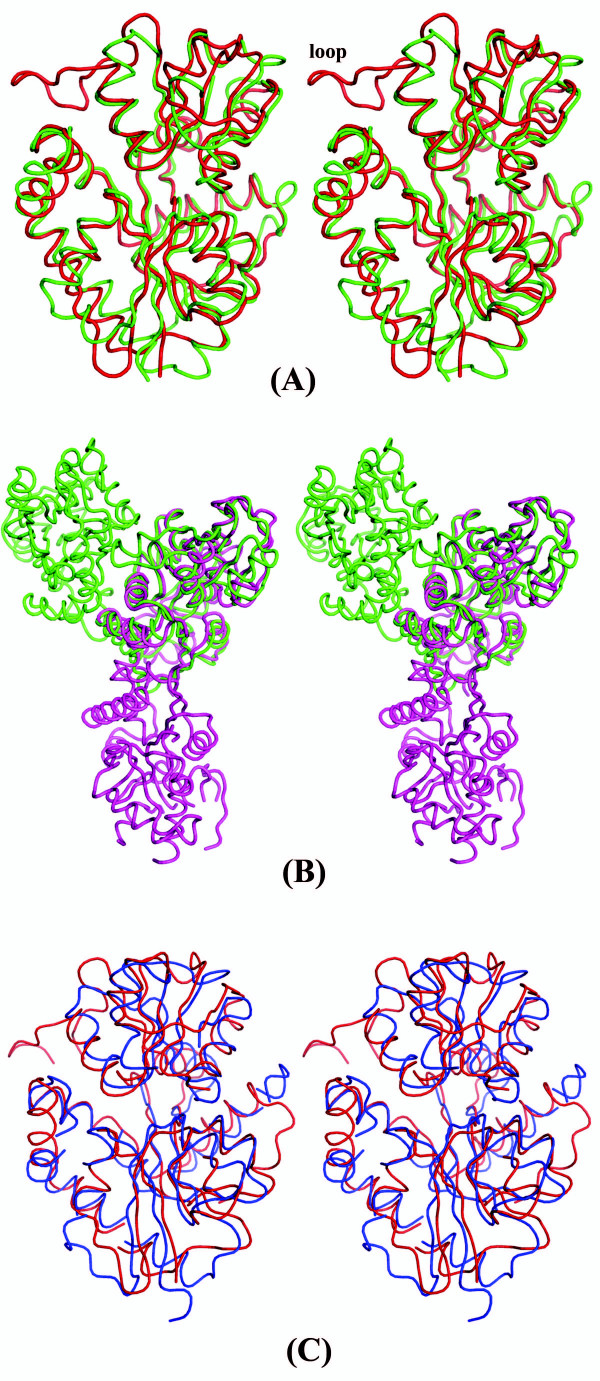
Stereoviews of (A) superposition of 10093b (red) and 10093f (green) monomers. The loop exclusive to 10093b is labeled. (B) superposition of 10093b (green) and 10093f (magenta) dimer. While 10093b is a dimer in solution, the dimerization of 10093f is due to crystal packing effect. (C) superposition of 10093b (red) and 1ZBM (blue) monomers.

The structure of 10093b was determined to 2.5Å resolution using Selenium-SAD (Table 1). The final refined model of 10093b contains 8 protomers, 18 sulfate ions, and 653 water molecules. The final refined model of 10093f (2.04Å resolution) contains 4 protomers and 579 water molecules. Despite low sequence identity (27%; Figure 1), the polypeptide chain structures are very similar (Figure 2). The two structures superimpose well with a rmsd of 1.9Å for 252 α-carbon atomic pairs, excluding a loop region between Val166 to Ser177 in 10093b that does not occur in 10093f (Figure 3A). This region is absent in all other sequences shown in Figure 1, except in *Thermus thermophilus *(Figure 1).

**Table 1:** Data collection, phasing and refinement statistics.

	**Se -SAD (10093b)**	**Se -SAD (10093f)**
**Cell dimensions**	*a *= 75.3,*b *= 139.4, *c *= 153.6; β = 92.8°	*a *= 75.8,*b *= 97.4, *c *= 86.6; β = 106.5°
**Space group**	P2_1_	P2_1_
**Data Collection Statistics**		
Wavelength (Å)	0.98	0.98
Temperature (K)	100	100
Resolution range	50.0-2.5	50-2.04
Outermost Shell (Å)	2.59-2.5	2.11-2.04
Unique reflections	107665 (9866)	76032(6908)
Completeness (%)	98.9(91.0)	98.8(89.7)
Mean I/σ(I)	11.1 (1.9)	16.8(2.1)
Redundancy	6.8(5.2)	4.6(3.9)
R_merge _^1^	0.095(0.40)	0.043(0.21)
**Phasing Statistics**		
Phasing power^2 ^(ano)	0.83	0.94
FOM^3^:	0.29	0.27
After density modification	0.93	0.92
**Refinement Statistics**		
No. of reflections (work)	100602	71996
No. of reflections (test)	3150	2264
^4^R_factor_/^5^R_free_	0.20/23.4	0.24/0.28
Resolution range (Å)	50.0-2.5	30.0-2.04
RMSD for bond length (Å)	0.006	0.006
RMSD bond angles (°)	1.35	1.4
<B-values>		
Main-chain (Å ^2^)	30.8	30
Side-chain (Å ^2^)	32.4	32.4
**Number of non-H atoms**		
No. of heteroatoms	90	0
No of water molecules	653	579

Values for the highest resolution shell are given within parentheses.

^1^R_merge _= Σ|I_i_-⟨I⟩|/Σ|I_i_| where I_i _is the intensity of the i^th ^measurement, and ⟨I⟩ is the mean intensity for that reflection.

^2^Phasing power and ^3^FOM (Figure of merit) as defined in SHARP.

^4^R_factor _= Σ||F_obs_|-|F_calc_||/Σ|F_obs_| where |F_calc_| and |F_obs_| are the calculated and observed structure factor amplitudes, respectively.

Notwithstanding the similarity of the two polypeptide chain folds, MR attempts with various 10093b-derived search models were not successful. Molecular replacement may have failed because of low sequence identity and/or due to the presence of multiple protomers within the asymmetric unit.

### Biologically active units

#### 10093b

Eight monomers A, B, C, D, E, F, G, and H found in the asymmetric unit form four tightly packed dimers (AB, CD, EF, and GH). Each dimer pair superimposes very well on the remaining three with rmsds = 0.74Å–0.78Å for all α-carbon atomic pairs. A dimer interface analysis performed using PDBSUM [3] demonstrated that each pair buries ~3,000Å^2 ^of solvent accessible surface area (12.3% of the total area), a value higher than would be found typically in interacting surfaces for a protein of this size [4]. At least 26 residues from each half of the dimer participate in intermolecular interactions. There are 6 direct hydrogen bonded interactions between the protein atoms across the dimer interface. Such a tight dimer interface suggests that the 10093b dimer is functional, which is supported by the results of analytical gel filtration (data not shown).

#### 10093f

Four monomers A, B, C, and D found in the crystallographic asymmetric unit occur as two dimers (AB and CD), albeit with an intermolecular packing arrangement that differs from that seen for 10093b. Each observed 10093f dimer pair buries ~1800 Å^2 ^of solvent accessible surface area, which is not thought to be significant for a protein of this size. Moreover, the gel-filtration analysis (data not shown) revealed that 10093f is a monomer in solution.

In the 10093b dimer, Glu172 from the atypical Val166-Ser177 loop makes one of the six hydrogen bonds present in the dimer interface while three other residues in the same loop contribute to van der Waals interactions. We suggest that the atypical loop found in 10093b may help support dimerization. The absence of this loop in 10093f may explain the monomeric solution behavior of this family member.

### Structure and sequence relationships and homology modeling

In an effort to annotate the function of these two related proteins, bioinformatics analysis with the experimental structures was performed using DALI [5]. For 10093b, a DALI search revealed only two structural matches with a Z-score greater than 10. The closest match, AF1704 from *Archaeoglobus fulgidus *(PDB ID: 1ZBM), a protein of unknown function belonging to Pfam DUF191 gave a Z-score of 19.8 with sequence identity of 15% and rmsd of 2.7 Å between 227 structurally equivalent α-carbon atomic pairs. Thus, structure determination of 10093b not only provided the first structural information for the Pfam family DUF178, it also documented unexpected structural similarity to a member of the DUF191 Pfam family that could not have been reliably predicted from sequence comparisons alone. A DALI search with 10093f revealed (excluding 10093b) significant matches (i.e., Z-score greater than 10) with AF1704 Z-score 19.4, as expected, and with a nitrate transport protein (PDB ID: 2G29) Z-score of 17.8 with sequence identity of 12% and rmsd between 252 structurally equivalent α-carbon atomic pairs of 3.3 Å. Stereoviews of the superimposed polypeptide chains of 10093b over 10093f-monomers, -dimers and 10093b over 1ZBM are presented in Figure 3A, 3B and 3C respectively.

A BLAST [6] search of Uniprot protein sequence database using the sequences of both 10093b and 10093f yielded 61 matches (sequence identities = 83-27%). Virtually all of the matches are identified as bacterial or archaeal hypothetical proteins. The three exceptions are as follows: a SAM-dependent methyltransferase from *Lactococcus lactis *(34% identity), leucyl-tRNA synthetase from *Xanthomonas campestris *(28% identity), D-alanine-D-alanine ligase from *Nitrosomonas europaea *(27% identity).

At the time of publication, the experimental structures of 10093b and 10093f were used as a template to compute homology models of 1133 proteins with related sequences with the Modweb server [7]. The 17 models out of 1133 had sequence identity of great than 30%.

### Active site/ligand binding site prediction

**Figure 4:**
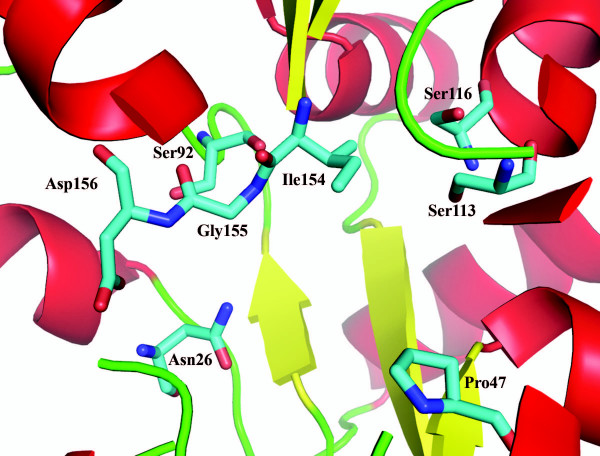
Zoom view showing putative binding site of 10093b. The β-strands (yellow ribbon), α-helices (red ribbon), and random coil (green ribbon), plus clustered conserved residues Asn26, Pro47, Ser92, Ser113, Ser116, Ile154, Gly155, and Asp156 from multiple sequence alignment shown in figure 1 (atom color coded stick figures; N-blue; C-sky blue; O-red). All residues numbers are labelled in black.

Active site/ligand binding site prediction performed using CASTp [8] revealed two major clefts on the surfaces of the 10093b and 10093f (10093b estimated areas: 699.3 Å ^2 ^and 277 Å ^2^). Further analysis of these surface features together with a multiple sequence alignment performed in ClustalW [9] and edited in BioEdit [10] (Figure 1) performed for 10093b reveals the presence of most of the conserved residues, including Asn26, Pro47, Ser92, Ser113, Ser116, Ile154, Gly155, and Asp156 (Figure 4). We suggest that this larger cleft represents the active site and/or ligand binding site for this functionally uncharacterized Pfam family.

### Prediction of putative function

10093b and 10093f belonging to DUF178 family have remarkable similarity to DUF191 protein family and a nitrate-binding protein (2G29). This similarity was not evident from sequence comparison alone. Moreover, the uncharacterized DUF191 family of proteins is annotated to be putative solute-binding protein. Our analysis of the surface clefts shows that the major cleft identified in 10093b is common to all of them, the size being nearly the same. The binding pocket of 10093b superposes well with that of nitrate-binding protein. Further analysis of the active site shows that the entry of this cavity is occupied by hydrophobic residues as in 2G29 [11]. In 10093b, the residual density in the difference Fourier showed a dumb bell shaped density and was modeled as water. This could very well be an unidentified ion. Based on the comparison of structures and binding sites, we predict that this protein could be a solute binding protein, though we cannot at this stage identify the nature of the ion. The weak homology implies that it may be a different kind of solute.

## Conclusion

The structure determination of 10093b and 10093f has revealed the unexpected similarity between DUF178 and DUF191 family of proteins suggesting that the information from three-dimensional structures along with the sequence information will predict the family of proteins of similar functions more reliably. We have predicted the protein to be a solute-binding protein based on structure and binding cavity. Fold and structural similarity among proteins with low sequence identity (less than 30%) is not uncommon. One prominent example would be proteins classified into different Pfams but with the same TIM barrel fold [12]. Amidohydrolases with low sequence homology have various substrate specificities and different enzymatic functions but all of them have similar fold. These belong to different Pfam families but are grouped together as a super family [12]. This also suggests that DUF178 and DUF191, though belonging to different Pfams of unknown functions, may be members of the same superfamily.

## Methods

### Protein production

#### 10093b

The target gene for 10093b was amplified using polymerase chain reaction (PCR) from *Deinococcus radiodurans *genomic DNA using a forward (ACCAACCATCACCCATCATCTAC) and a reverse (CTGCTTCCTCACGCGCTCCGAAG) primer.

#### 10093f

The target gene was amplified similar to 10093b from *Streptomyces coelicolor *genomic DNA using Forward (GATAATAGCCGTACCCGCCC) and a reverse (CAGGTTTCAGCAACTCAACCTTG) primer.

The amplified genes of both 10093b and 10093f were gel purified and cloned into pSGX3 (BC) vector designed to express the protein of interest with a C-terminal hexa-histidine tag to facilitate easy and high yield purification. Protein expression/purification utilized previously published protocols [13]. For 10093b a yield of 22 mg was obtained from 3L culture, whereas for 10093f the yield was 91 mg from 2L culture.

### Crystallization, data collection and structure determination

#### 10093b

Native and Se-Met crystals of 10093b were grown at 20°C via the sitting drop vapor diffusion method (crystallization drop contained 2 μL of 22 mg/mL protein plus 2 μL of reservoir solution containing 25% (w/v) PEG 3350, 0.1 M Bis-Tris pH 5.5, 0.2 M NH_4_SO_4_, and 1 μL of 0.1 M TCEP hydrochloride). Rod shaped crystals with dimensions 0.5 × 0.02 × 0.02 mm^3 ^appeared after two days. Crystals were flash frozen in liquid nitrogen following addition of 20% ethylene glycol to the mother liquor. Diffraction data were collected at beamline X12C, National Synchrotron Light Source (NSLS), Brookhaven National Laboratory and processed using HKL2000 [14]. Both crystals belong to monoclinic space group P2_1_. The calculated Matthews coefficient is 3.1 Å ^3^/Da (solvent content 59.4% by volume), assuming eight molecules/asymmetric unit. All 32 possible selenium sites were found by SHELXD [15] using the peak data collected at the selenium absorption edge (λ = 0.98 Å). Phase refinement and density modification were performed with SHARP [16]. The final improved electron density map after density modification was of high quality and allowed automated model building of about 85% of the polypeptide chain with ARP/wARP [17]. The remainder of the polypeptide chain was built manually using both Sigma-weighted 2|Fo|-|Fc| difference Fourier map from CNS and experimental electron density map from SHARP using O [18]. The structural model was refined to convergence using CNS [19]. For R_free _calculation 3% of randomly selected data was excluded from the refinement. The Ramachandran plot calculated using PROCHECK [20], shows 89.9% residues in the most favorable region. Arg173 in chain G in the loop region occurs in disallowed region, probably because of poor resolution of the electron density. The structures of individual 10093b protomers found in the asymmetric unit were highly similar to one another (pairwise root-mean-square-deviations or rmsds = 0.74Å–0.78Å).

#### 10093f

Rod shaped crystals (dimensions; 0.3 × 0.02 × 0.02 mm^3^) similar to 10093b were obtained for native protein in 25% (w/v) PEG 3350, 0.1 M Bis-Tris pH 5.5, and 0.2 M MgCl_2 _and 10% Jeffamine and native diffraction data were collected at beamline X12C. As sequence identity with 10093b was ~27%, molecular replacement (MR) was attempted but did not yield meaningful phases. Accordingly, SeMet protein was crystallized using similar condition and Se-SAD diffraction data were collected at beamline X29A (NSLS). 10093f crystals grow in monoclinic P2_1 _space group with four molecules/asymmetric unit. All possible 16 selenium sites were found by SHELXD [15]. Phase refinement and density modification were performed in SHARP [16]. The final improved electron density map after density modification was of high quality and allowed automated model building of about 85% of the polypeptide chain with ARP/wARP [17]. The remainder of the polypeptide chain was built manually using O [18], and the resulting structural model was refined to convergence using CNS [19]. The Ramachandran plot calculated using PROCHECK [20] shows 89.1% residues in the most favorable region. Five residues (Ala146 and Met 84 in chain A, Met84 in chain B, Met84 and Leu136 in chain C and Met84 in chain D) occur in disallowed region, probably because of poor resolution of the electron density. The structures of individual 10093f protomers found in the asymmetric unit were highly similar to one another (pairwise rmsds = 0.76–0.78Å).

Data collection, phasing and refinement statistics for both structures are provided in Table 1. The coordinates and structure factors of both structures have been deposited with the Protein Data Bank (10093b: 2I6E; 10093f: 2NXO).

## Competing interests

The author(s) declares that there are no competing interests.

## Authors' contributions

RT carried out crystallographic studies and prepared the manuscript. SKB helped in preparing the manuscript. SS helped in determining and analyzing the structure.
